# Entomological and parasitological impacts of indoor residual spraying with DDT, alphacypermethrin and deltamethrin in the western foothill area of Madagascar

**DOI:** 10.1186/1475-2875-13-21

**Published:** 2014-01-14

**Authors:** Jocelyn Ratovonjato, Milijaona Randrianarivelojosia, Maroafy E Rakotondrainibe, Vaomalala Raharimanga, Lala Andrianaivolambo, Gilbert Le Goff, Christophe Rogier, Frédéric Ariey, Sébastien Boyer, Vincent Robert

**Affiliations:** 1 Institut Pasteur de Madagascar, Ambatofotsikely, Antananarivo 101, BP 1274, Madagascar; 2 Ministère de la Santé, Programme National de Lutte contre le Paludisme, Androhibe, Antananarivo 101, BP 8169, Madagascar; 3 MIVEGEC (Maladies Infectieuses et Vecteurs: Ecologie, Génétique, Evolution et Contrôle) UMR IRD 224, CNRS 5290, UM1, UM2, Centre IRD France-Sud, BP 64501, Montpellier Cedex 5, 34394, France; 4 Unité de recherche Génétique et génomique des insectes vecteurs, Institut Pasteur de Paris, 25-28 rue du Dr Roux, Paris, 75015, France

**Keywords:** Indoor residual spraying, DDT, Pyrethroids, *Anopheles funestus*, *Anopheles arabiensis*, *Anopheles mascarensis*, *Plasmodium falciparum*, Malaria

## Abstract

**Background:**

In Madagascar, indoor residual spraying (IRS) with insecticide was part of the national malaria control programme since the middle of the twentieth century. It was mainly employed in the highlands and the foothill areas, which are prone to malaria epidemics. Prior to a policy change foreseeing a shift from DDT to pyrethroids, a study was carried out to assess the entomological and parasitological impacts of IRS in areas with DDT or pyrethroids and in areas without IRS.

**Methods:**

The study was carried out from October 2002 to February 2005 in three communes of the western foothill area of Madagascar. Two communes received IRS with DDT in February 2003, then IRS with pyrethroids (alphacypermethrin or deltamethrin) in February 2004. The third commune remained untreated. Mosquitoes were collected at night using human landing catches and early in the morning in resting places. Blood smears were obtained from schoolchildren and microscopically examined for *Plasmodium* presence.

**Results:**

In total, 18,168 human landing mosquitoes and 12,932 resting anophelines were collected. The *Anopheles* species caught comprised 10 species. The main and most abundant malaria vector was *Anopheles funestus* (72.3% of human-seeking malaria vectors caught indoors). After IRS had taken place, this species exhibited a lower human biting rate and a lower sporozoite index. Overall, 5,174 blood smears were examined with a mean plasmodic index of 19.9%. A total of four *Plasmodium* species were detected. Amongst tested school children the highest plasmodial index was 54.6% in the untreated commune, compared to 19.9% in the commune sprayed with DDT and 11.9% in the commune sprayed with pyrethroid. The highest prevalence of clinical malaria attacks in children present at school the day of the survey was 33% in the untreated commune compared to 8% in the areas which received IRS.

**Conclusion:**

In terms of public health, the present study shows (1) a high efficacy of IRS with insecticide, (2) a similar efficacy of DDT and pyrethroid and (3) a similar efficacy of alphacypermethrin and deltamethrin. The use of IRS with DDT and pyrethroid greatly decreased the vector-human contact, with an associated decrease of the plasmodial index. However malaria transmission did not reach zero, probably due to the exophilic host-seeking and resting behaviours of the malaria vectors, thus avoiding contact with insecticide-treated surfaces indoors. The study highlights the strengths and weaknesses of the IRS implementation and the need for complementary tools for an optimal vector control in Madagascar.

## Background

In Madagascar, an intensive campaign to eliminate malaria begun in 1949 by applying indoor residual spraying (IRS) with DDT insecticide [1]. This vector control activity was intensified during the Global Malaria Eradication Campaign between 1955 and 1970 [2]. Implemented successfully throughout the entire country, in association with chloroquine treatment for malaria patients at a community level [3], these interventions led to a significant decrease in the number of confirmed cases [4]. DDT was sprayed on the interior and exterior walls of houses in a general campaign that was named 'Operation de Pulvérisation Intradomiciliaire d’Insecticides’ (OPID). A temporary elimination of malaria was noted at the beginning of the 1970s. During the same period, *Anopheles funestus*, the primary malaria vector disappeared from the Central Highlands area, except in three districts [5]. The abandonment of OPID in 1979, the discontent with health structures and the re-invasion of the Central Highlands by *An. funestus* led to a malaria outbreak in 1986 [4,6,7]. In response, a vector control programme was implemented from 1993 to 1998. Here DDT was used for IRS in the Central Highlands at altitudes between 1,000 and 1,500 m that delimit the area prone to malaria epidemics. This campaign was locally known as 'Campagne d’Aspersion Intradomiciliaire d’Insecticide’ (CAID) [8]. In parallel, treatment with chloroquine was incorporated in areas where a high risk of malaria outbreaks was detected [9]. From 1999 until 2005 CAID were replaced by more selective operations in restricted areas above 900 m. The Malagasy National Malaria Control Programme revised its strategy and pyrethroid insecticides replaced DDT in 2005 for IRS in the highlands.

At the end of the 1960s all DDT use was banned in the majority of developed countries but exceptions were made for countries needing essential public health interventions. However, its low cost and persistence make DDT a very useful insecticide and it is thus still used for IRS in resource-poor countries. DDT is not easily biodegradable and there is evidence of adverse effects to human health, so it was widely agreed that a replacement insecticide with the same cost-effectiveness has to be found [10-12]. Madagascar, like other countries with unstable and epidemic-prone areas was authorized to use DDT for disease vector control until the availability of an equally effective alternative insecticide [13]. The pyrethroid class of insecticides was derived from the natural pyrethrum pesticide produced by Chrysanthemum flowers. Produced by the chemical industry, pyrethroids are described as an alternative to DDT for IRS and the only insecticides recommended by the WHO for impregnating bed nets [14,15].

The emergence of resistance of several malaria vectors to insecticides threatens successful malaria vector control in some countries [16]. New tools were suggested for reducing malaria transmission, but nevertheless, chemical insecticides are still essential [17,18], and in most malaria-endemic countries, especially sub-Saharan Africa, IRS remains an important method of fighting endophilic malaria vectors [19,20].

This article compiles results dispersed in grey literature of the Institut Pasteur de Madagascar [21-23]. It reports the effectiveness of four regimens of IRS for malaria vector control in the Malagasy Central Highlands: absence of IRS, IRS with DDT or alternatively with two pyrethroids (deltamethrin and alphacypermethrin). The strengths and weaknesses of IRS and the need for complementary tools for effective malaria vector control are discussed.

## Methods

### Study area

**Figure 1:**
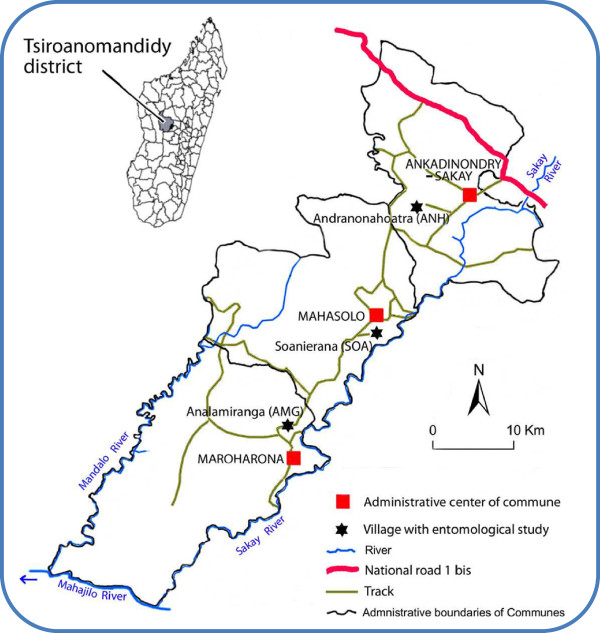
The study area in Madagascar.

**Figure 2:**
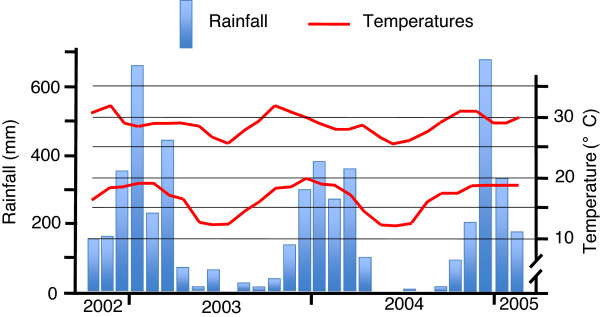
Minimal and maximal mean temperatures and rainfall recorded at Mahasolo, from October 2002 to February 2005.

The study area is located in the district of Tsiroanomandidy in the western foothills of the Malagasy Central Highlands, more precisely in the western and right bank of the Sakay River (Figure 1). The entomological study was conducted in three *communes*, Andranonahoatra (ANH) (19°00’34”S; 46°25’21”E; 920 m a.s.l.), Soanierana (SOA) (19°08’42”S; 46°25’26”E; 900 m), and Analamiranga (AMG) (19°14’35”S; 46°16’22”E; 885 m). In strait line, the distances ANH-SOA and SOA-AMG are 14 and 16 km, respectively. The Mahasolo meteorological station, about in the middle of the study area, recorded annual rainfalls of 2,022 and 2,142 mm in 2003 and 2004, respectively, and 105 rainy days for each year. Minimal and maximal mean temperatures were 12.0°C and 32.0°C for the whole study (Figure 2). The villages ANH, SOA and AMG have 1,002, 1,274 and 900 inhabitants, respectively. Most are farmers with rice as their main activity/resource and maize, manioc and peanuts as their secondary crop. They declared not to sleep under a mosquito net, except one couple in SOA and three couples in AMG. Zebus in the three villages were 400, 160 and 390, respectively; they stay at night in parks located inside villages. The houses were built by mud with thatched or iron sheet roofing and with or without flooring.

The parasitological study was conducted in schools of the communes Ankadinondry-Sakay, Mahasolo and Maroharona, in which are located the villages ANH, SOA and AMG, respectively. As most of surrounding areas, the communes Ankadinondry-Sakay and Mahasolo were DDT sprayed annually in the nineteen’s, but not in 2002. The commune Maroharona was not sprayed at all during the three decades previous to the present survey [24].

In this area, like in the western part of the Malagasy highlands at similar altitudes, malaria is endemic. The transmission is performed by one major vector, *An. funestus*, and two secondary vectors, *Anopheles arabiensis* and *Anopheles mascarensis*, at the exclusion of any other species [25-28]. As in most of Madagascar, these anophelines exhibit a strong tendency to exophily, both for biting and resting behaviour, and zoophily for bloodmeals [24,29]. The zoophily of *An. funestus* greatly contrasts with the whole of tropical Africa where this species is mainly anthropophilic [30]. It has been suggested that DDT house-spraying campaigns performed in the Malagasy highlands might had selected for behavioural change on a genetic background including zoophily in the behavioural repertoire [31]. The prevalence of blood parasite in children is known to greatly vary between near zero and 50%, depending of various parameters such as the season, the existence of IRS, and the access to anti-malarial drugs and health facilities [24,32-34].

### Study design

**Figure 3:**
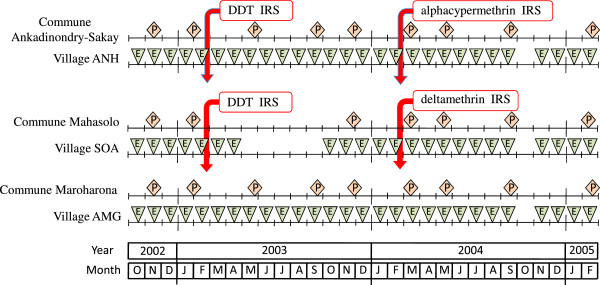
**Chronogram of studies performed in Ankadinondry-Sakay commune, including the village Andranonahoatra (ANH), Mahasolo commune, including the village Soanierana (SOA), and Maroharona commune, including the village Analamiranga (AMG).** The parasitological and entomological surveys are noted 'P’ and 'E’, respectively. Indoor residual spray (IRS) with DDT and pyrethroids were performed in February 2003 and February 2004, respectively; the Maroharona commune remained untreated.

In the two communes of Ankadinondry-Sakay and Mahasolo the study was conducted with three sequences of activities (pre-treatment, IRS with DDT and IRS with pyrethroids). The commune Maroharona remained untreated. Entomological observations were repeated monthly and parasitological surveys every three-month period (Figure 3). The study was carried out during 29 months from October 2002 to February 2005. The Malagasy Health and Education Authorities approved the entire protocol.

### Insecticide spraying

IRS was carried out in a routine way by the Malagasy National Malaria Control Programme team with community participation and agreement. Three insecticides were used: 75% pure DDT wettable powder sprayed at 2 g of active ingredient (a.i.)/sq m, an alphacypermethrin (pyrethroid) wettable powder (Fendona®) sprayed at 30 mg a.i./sq m and a deltamethrin (pyrethroid) WG 250 wettable powder (K-Othrine WG250®) sprayed at 25 mg a.i./sq m. The insecticides used were approved by WHO. A first round of IRS was carried out with DDT at the end of February 2003 in the two communes Ankadinondry-Sakay and Mahasolo and a second round at the end of February 2004 with deltamethrin in the commune Mahasolo and with alphacypermethrin in the commune Ankadinondry-Sakay.

### Entomological investigations

Entomological study was performed routinely, once a month during the whole study, in the three villages ANH, SOA and AMG, except in SOA during the five months May to September 2003 and in the three villages in October 2004 (Figure 3).

#### Human landing catch

Monthly mosquito collections were carried out from 06.00 p.m. to 06.00 a.m. when landing on human beings inside and outside houses, according to WHO procedures [35]. Two consecutive nights of mosquito collections were carried out per village and per month, in four houses, with four mosquito collectors (two inside and two outside houses) per night. Consequently, a total of 16 human-nights were performed per month in each village and the total of human-nights was 1,384 for the whole study.

#### Collection of resting mosquito

Resting mosquitoes were collected indoors and outdoors. Endophilic resting mosquitoes were collected mornings from 10 bedrooms per village and per month. The technique involved spraying a non-permanent insecticide (with aerosols containing pyrethroids) in a room where all exits were blocked as effectively as possible. Mosquitoes killed or simply stunned by the effect of insecticide fell on white sheets placed on the floor or furniture. All mosquitoes at rest in the room were collected. The total of bedroom-nights was 660 for the whole study. Exophilic resting mosquitoes were collected early after sunrise in two to five (mean 3.7 for the whole study) Muirhead-Thomson’s pit shelters per village [36] and in various resting places such as animal shelters and cart sheds. Any exophilic mosquitoes that were found were drawn up with a sucking tube. A total of 166 pit-nights and a non-registered number of other resting places were visited during the whole study.

#### Mosquito identification

The mosquitoes were morphologically identified using taxonomic keys [25,30]. Legs or wings of mosquitoes from the *An. gambiae* complex were used for PCR identification [37]. Extracted DNA from these mosquito parts was added to the reaction mixture. The amplification was done under the following conditions: 5 min at 94°C followed by 30 cycles of 1 min at 94°C, 50 sec at 50°C and 50 sec at 72°C, with a final elongation step during 5 min at 72°C. The expected sizes of the fragments were 315, 390 and 464 base pairs for *An. arabiensis*, *An. gambiae* and *An. merus*, respectively. These three species were previously recorded in Madagascar [38]. *Anopheles funestus*, *An. arabiensis* and *An. mascarensis* females were examined for parity, anthropophily and infectivity.

#### Ovary dissection

Ovaries from malaria vector specimens caught on human landing were dissected to determine parity rate, a proxy of the longevity, by the observation of the degree of ovarian tracheole coiling, based on microscopic observation immediately after collection [39].

#### Blood feeding patterns of resting mosquito

Engorged resting anopheline females were soon dissected and their abdomens were squashed on filter paper then dried with silica gel in the field then conserved at 4°C in laboratory. Blood spots were tested with ELISA with antibodies for human, bovid, pig and dog according to [40]. The absorbance was recorded at 405 nm and 620 nm with an ELISA plate reader (Biotek EL 800).

#### Estimation of the mosquito infection

Head and thorax were dissected and processed by ELISA in a first step, using monoclonal antibodies against *Plasmodium* circumsporozoite protein (CSP) and, if positive, in a second step, monoclonal antibodies against *Plasmodium falciparum*, *P. malariae*, *P. vivax* 210 or *P. vivax* 247 in order to determine the *Plasmodium* species [41,42]. Results on *P. malariae* were not presented, obviously spoiled by numerous false positives (see discussion). ELISA was not performed using antibodies against *P. ovale*.

#### Entomological indicators

The daily biting rate, an estimation of man-mosquito contact, was calculated as the ratio of the number of mosquitoes caught landing on human subjects to the number of human-nights. The endophagic rate was estimated from mosquitoes sampled by human landing collection indoor / (indoor + outdoor). The anthropophilic rate was calculated as the number of mosquitoes fed on human beings divided by the number the gorged mosquitoes tested for blood meal analysis; following [43-45], mixed human blood meals were included in this calculation, but mosquitoes with unidentified blood meal sources were excluded. The parity rate was the ratio of the number of parous females to the number of parous + nulliparous females; it was calculated using the biting females [46]. The infectivity rate was the ratio of the number of mosquitoes positive by ELISA for *P. falciparum* + *P. vivax* to the number of examined mosquitoes. The entomological inoculation rate (EIR) was the product of the biting rate and infectivity rate. This calculation was performed daily taking the anopheline vector species one by one, then summed in the final step. Since dwellers spend all nights in their houses, the human biting rate was considered indoors for EIR calculation.

### Parasitological and clinical investigations

Parasitological surveys were performed about every three months from November 2002 to February 2005, in the schools of the two communes Mahasolo and Ankadinondry-Sakay, except during the months May to September 2003 in Ankadinondry-Sakay (Figure 2). Informed consent for the participation of the schoolchildren was obtained from their legal guardians. Every transversal survey concerned at least 400 schoolchildren present at school the day of the survey in the primary schools of the commune Ankadinondry-Sakay (Ecole Publique Primaire (EPP) Andranonahoatra and EPP Soamihary), the commune Mahasolo (EPP Soanierana, EPP Maevarano and EPP Ambohimandroso Bemasoandro) and the commune Maroharona (Sainte-Thérèse Analamiranga, EPP Analamiranga and EPP Ampasipotsy). Any child was questioned for symptoms related to malaria; if the presence of malaria was suspected, the child was treated in agreement with the national health policy.

A drop of blood was collected from the fingertip and thick and thin blood smears were prepared and giemsa stained. Thick blood film readings were standardized and quality control was performed. Parasite densities were determined by counting the number of asexual parasites per 200 white blood cells (WBCs) or per 500 WBCs if the count was less than 10 parasites/200 WBCs. Assuming a WBC count of 8,000/mm^3^, the sensitivity threshold was estimated at 16 parasites/mm^3^. The thin smear was used if plasmodial species determination was doubtful in thick smear. The proportion of people who are infected with any *Plasmodium* was expressed as the plasmodial index. The total of blood smears was 5,174 for the whole study.

Fever was defined as an axillary temperature ≥37°C. Malaria attack was defined as an association of fever and asexual parasite of *Plasmodium* spp. in blood smear; no minimal parasite density was assigned to define a malaria attack, in line with previous studies performed in this area [47].

### Data processing

Entomological, demographic, parasitological and clinical data were entered in an Excel database (Additional file 1: Table S1 and Additional file 2: Table S2) and analysed using STATA 11.0 statistical software (StataCorp LP, College Station, TX: Stata Corporation, 2009). The base line data were provided from October 2002 to February 2003, *i.e.* during the period previous to any IRS.

The numbers of mosquitoes caught on humans (indoors and outdoors) and at rest (in bedrooms and in pit shelters) were analysed in population-averaged negative binomial regression models using a generalized estimating equations (GEE) approach taking into account an exchangeable within-village and survey correlation structure. The rates of endophagy, parity, anthropophily and infectivity of the mosquitoes were analysed in population-averaged logistic regression models using a GEE approach and taking into account an exchangeable within-site and survey correlation structure. The villages, the type of IRS (DDT, alphacypermethrin and deltamethrin), the periods (*i.e.* trimesters or years of survey) and the catching location (indoors and outdoors) were initially introduced in full regression models. The interactions between the effect of periods, the catching location and the type of IRS were tested. A backward stepwise selection procedure was applied to retain the significant (P < 0.05) independent variables.

Prevalence rates of plasmodial infections and clinical malaria attacks were estimated with their 95% confidence intervals (CI) taking into account the interdependence of observations made in the same site and during the same survey, and were analyzed as dependent variables in population-averaged logistic regression models using a GEE approach and taking into account an exchangeable within-commune and survey correlation structure. The communes, the type of IRS (DDT, alphacypermethrin and deltamethrin), the periods, the age and the sex were initially introduced in a full logistic regression model. A backward stepwise selection procedure was applied to retain the significant (P < 0.05) independent variables. The statistical quality of the final models was assessed by looking at the adequacy between observed and predicted prevalences.

The differences between villages/communes, periods, type of catching and type of IRS were tested by the Wald test, and 95% CI of adjusted odds ratio (OR) or incident relative risk (IRR) were calculated. The data processing takes into account the gap previously mentioned in the data collection due to problems in funds availability in the Mahasolo commune during the five-month period, March-September 2003 (Figure 2). The data collected during this period were excluded from the regression analysis.

## Results

### Mosquitoes and malaria transmission

#### Mosquito species composition and relative abundance

A total of 8,001 *An. gambiae s.l.* was collected with various methods during the whole study. A sample of 2,287 was PCR identified and unambiguously assigned to a single species, *An. arabiensis*. In human landing catches, 18,168 mosquitoes were collected (Table 1). *Anopheles* was the most abundant mosquito genus collected with 13,427 (73.9%) as against 3,979 *Culex* (21.9%), 676 *Mansonia* (3.7%), 76 *Aedes* (0.4%) and 10 *Coquilletidia* (0.1%). Three species of potential malaria vectors, *An. funestus*, *An. arabiensis* and *An. mascarensis*, accounted for 30.0% of the mosquitoes and 40.6% of anophelines. Using the methods for collection of resting mosquitoes, 12,932 of these three anopheline species were collected (Table 2). After IRS, no change was noted in species composition of mosquitoes in the different villages.

**Table 1:** Number of mosquitoes collected by human landing catch indoors and outdoors

**Village**	**Andranonahoatra**		**Soanierana**		**Analamiranga**		**Total**
	**Indoors**	**Outdoors**	**Indoors**	**Outdoors**	**Indoors**	**Outdoors**	
*An. coustani*	103	308	451	1,901	100	991	3,854
*An. funestus*	72	132	69	151	1,035	2,374	3,833
*An. squamosus-cydippis*	25	109	364	1,475	27	191	2,191
*An. arabiensis*	98	260	200	758	47	251	1,614
*An. mascarensis*	18	108	26	185	61	1,022	1,420
*An. maculipalpis*	8	56	23	140	10	131	368
*An. rufipes*	2	10	9	20	3	29	73
*An. pharoensis*	1	5	5	16	0	4	31
*An. flavicosta*	1	0	1	3	3	19	27
*An. pretoriensis*	0	4	0	0	3	9	16
*Culex* spp.	139	506	422	1,678	274	960	3,979
*Mansonia uniformis*	26	90	61	234	50	215	676
*Aedes* spp.	0	4	4	32	8	28	76
*Coquillettidia grandidieri*	1	6	0	2	1	0	10
Total (%)	494 (2.7)	1,598 (8.8)	1,635 (9.0)	6,595 (36.3)	1,622 (8.9)	6,224 (34.3)	18,168 (100.0)

**Table 2:** Number of resting anophelines and percentage of catch per village and resting location

**Village**	**Andranonahoatra**		**Soanierana**		**Analamiranga**		**Total (%)**
	**Bedroom resting**	**Outdoor resting**	**Bedroom resting**	**Outdoor resting**	**Bedroom resting**	**Outdoor resting**	
*An. funestus*	355	2,537	58	2,459	49	930	6,388 (49.4)
*An. arabiensis*	134	939	151	582	1,700	2,360	5,866 (45.4)
*An. mascarensis*	6	200	1	124	12	335	678 (5.2)
Total (%)	495 (3.8)	3,676 (28.4)	210 (1.6)	3165 (24.5)	1,761 (13.6)	3,625 (28.0)	12,932 (100.0)

#### Biting rate

**Figure 4:**
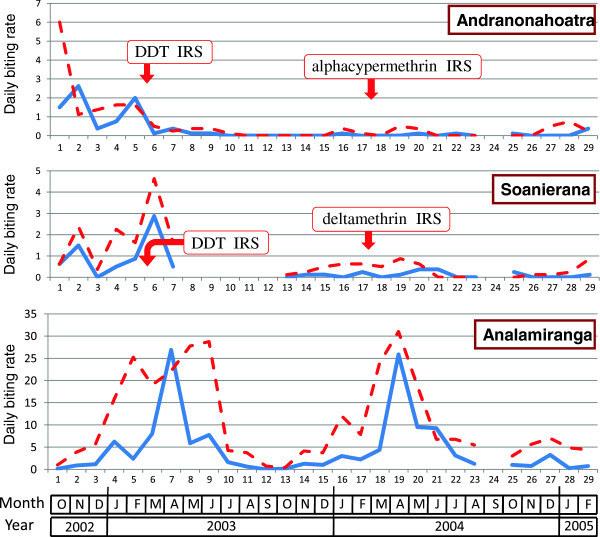
**Daily biting rate of*An. funestus*(number of bites per human and per night) in the villages of Andranonahoatra, Soanierana and Analamiranga.** Mosquitoes were collected by human landing indoors (blue line) and outdoors (red dashed line). Indoor residual spray (IRS) with DDT and pyrethroids were performed in February 2003 and February 2004, respectively; the Maroharona commune remained untreated.

**Figure 5:**
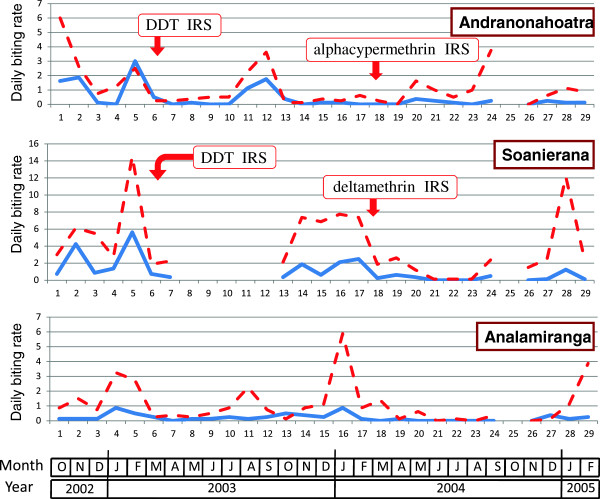
**Daily biting rate of*An. arabiensis*(number of bites per human and per night) in the villages of Andranonahoatra, Soanierana and Analamiranga.** Mosquitoes were collected by human landing indoors (blue line) and outdoors (red dashed line). Indoor residual spray (IRS) with DDT and pyrethroids were performed in February 2003 and February 2004, respectively; the Maroharona commune remained untreated.

**Figure 6:**
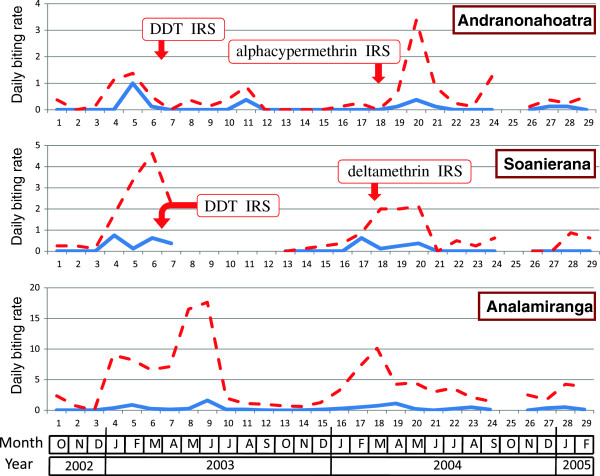
**Daily biting rate of*An. mascarensis*(number of bites per human and per night) in the villages of Andranonahoatra, Soanierana and Analamiranga.** Mosquitoes were collected by human landing indoors (blue line) and outdoors (red dashed line). Indoor residual spray (IRS) with DDT and pyrethroids were performed in February 2003 and February 2004, respectively; the Maroharona commune remained untreated.

The evolution of daily biting rate of *An. funestus*, *An. arabiensis* and *An. mascarensis* is presented Figures 4, 5 and 6. Overall, these species accounted for 72.3%, 21.2% and 6.5% of malaria vectors biting indoors and 50.7%, 24.2% and 25.1% of malaria vectors biting outdoors, respectively.

During the first, third and fourth trimesters post-DDT IRS, compared to the pre-IRS period and controlling for the variation of the anopheline aggressiveness in Analamiranga, the biting rate for the sum of the three malaria vectors was 83% to 94% (depending of trimester) lower in Andranonahoatra (0.06 < IRR < 0.17, 0.44 < P < 0.0001), suggesting an impact of IRS with DDT. Similar observations were done during the first and second trimesters post-pyrethroid IRS in both Soanierana and Andranonahoatra (0.16 < IRR < 0.05, 0.008 < P < 0.0001) suggesting an impact of IRS with pyrethroids. This reduction was also observed in the third trimester post-pyrethroid but without significant difference.

Considering *An. funestus* alone, and considering successively each of the four trimesters post-pyrethroid IRS, compared to the pre-IRS period and controlling for the variation of the aggressiveness in Analamiranga, no significant difference was observed between the two sprayed villages, suggesting a similar impact (1) of DDT in each of the two sprayed villages, and (2) of alphacypermethrin and deltamethrin. Considering *An. arabiensis* alone, similar observations were done in trimesters 1, 3 and 4 post-pyrethroid IRS. This was not observed in trimester 2, with significant lower IRR in Soanierana than in Andranonahoatra (IRR=0.08 and IRR = 13.0, respectively, Chi square = 8.11, df = 1, P = 0.0044), but this is probably due to the quasi absence of *An. arabiensis* during this trimester in Analamiranga (Figure 5). The biting rate of *An. mascarensis* indoors was very low (daily mean =0.13) and its variation was not statistically analysed.

The aggressiveness outdoors was consistently higher than indoors. During the whole study, the endophagic rate for biting activity was 30.5%, 21.3% and 8.0% for *An. funestus*, *An. arabiensis* and *An. mascarensis*, respectively. Overall, the risk for being bitted by anopheline vectors was much higher outdoors than indoors (IRR = 3.78, P < 0.0001).

#### Density of indoor- and outdoor-resting anophelines

The three malaria vectors *An. funestus*, *An. arabiensis* and *An. mascarensis* represented 49.4%, 45.4% and 5.2% of resting anophelines (Table 2). The number of bedrooms used for pyrethrum spray catches was 280, 230 and 280 in Andranonahoatra, Soanierana and Analamiranga, respectively; therefore the density per room of resting vectors was 1.77, 0.91 and 6.29, respectively, suggesting a strong impact of IRS in lowering the densities indoors. Such difference was not observed in vectors resting outdoors, an observation that can be related to the fact that insecticide was exclusively sprayed indoors.

#### Blood meal identification

Among the mosquitoes collected at rest, a total of 4,514 gorged anophelines was tested for blood feeding patterns during the whole study, and the blood source was identified for 4,396 (97.4%). The blood of human, bovid, pig and dog was present in 14.2%, 85.1%, 1.6% and 1.7%, respectively (total = 103.7% due to 118 mixed blood meals).

The anthropophilic rate (AR) was 28.8%, 2.5% and 1.6% in *An. funestus*, *An. arabiensis* and *An. mascarensis*, respectively. AR was consistently higher in mosquitoes resting indoor in bedrooms than resting outdoor (Table 3), in line with the usual habit for human beings to sleep inside house and conversely for zebus to spend the night outdoors in enclosures within the village perimeter. The incident relative risk (IRR) for a blood meal of *An. funestus* to be taken on human being was 0.06 outdoors compared to 1 indoors (P < 0.0001), whatever the period, before or after any IRS.

**Table 3:** Number of resting anophelines and anthropophilic rate (percentage of mosquitoes fed on humans), per village and resting location

**Village**	**Andranonahoatra**		**Soanierana**		**Analamiranga**		**Total**
**Resting site**	**Bedroom resting**	**Outdoor resting**	**Bedroom resting**	**Outdoor resting**	**Bedroom resting**	**Outdoor resting**	
*An. funestus*	61 (39%)	269 (10%)	95 (65%)	192 (3%)	692 (50.3%)	655 (15.0%)	1,964 (28.8%)
*An. arabiensis*	204 (5%)	850 (1.7%)	42 (5%)	605 (0.7%)	22 (14%)	402 (5%)	2,125 (2.5%)
*An. mascarensis*	2/3*	83 (1%)	0/1*	64 (2%)	0/9*	147 (1%)	307 (1.6%)
Total (%)	268	1,202	138	861	723	1,204	4396

*Anthropophilic rate not calculated when No. < 25.

During the whole study, the mean AR of *An. funestus* was lower in Andranonahoatra and Soanierana, compared to Analamiranga (27%, 27% and 48%, respectively) (Table 4). Considering the AR of *An. funestus* during the pre-IRS period, the differences were statistically significant between villages (OR = 0.31, P < 0.001 for Andranonahoatra; OR = 0.44, P = 0.024 for Soanierana, compared to OR = 1 for Analamiranga). A significant higher AR of *An. funestus* was observed during the IRS period with DDT compared to the pre-IRS period (OR = 7.72, P < 0.001). This unexpected result was mainly due to mosquitoes collected indoors in March 2003 in Soanierana. No significant variation in AR of *An. funestus* was observed during the IRS period with pyrethroids compared to the pre-IRS period. These results suggest that IRS did not reduce the AR of *An. funestus* collected at rest. Such analysis failed to test the impact of IRS on ARs of *An. arabiensis* and *An. mascarensis* due to their very low ARs, especially in Analamiranga, and the impossibility to adjust the models because of the absence of detection of blood meals on humans during the baseline period in some villages.

**Table 4:** **Number of *Anopheles funestus *** **and anthropophilic rate (AR), per period before and after indoor residual spraying (IRS) with DDT or pyrethroid (pyr)**

**Village**	**Andranonahoatra**		**Soanierana**		**Analamiranga**	
**Period**	**No.**	**AR (%)**	**No.**	**AR (%)**	**No.**	**AR (%)**
Oct 02-Feb 03 (pre-IRS)	151	27	123	27	237	48
Mar 03-Feb 04 (IRS DDT)	97	6	96	34	553	24
Mar 04-Feb 05 (IRS pyr)	82	6	68	3	557	35
Overall	330	15.7	287	23.7	1,347	33.1

#### Parous rate

The mean parous rate (PR) of *An. funestus*, *An. arabiensis* and *An. mascarensis* was 88.6%, 64.9% and 87.1%, respectively. In the untreated village of Analamiranga, the PR of the three vectors was similar in human landing catches indoors and outdoors, like in resting sites indoors and outdoors (Table 5).

**Table 5:** Number of malaria vectors and parous rate (PR) per method of collection, by human landing catches indoors and outdoors and by aspiration of mosquitoes at rest in bedrooms and pit shelters

	**Village**	**Andranonahoatra**		**Soanierana**		**Analamiranga**		**Total**	
		**No.**	**PR (%)**	**No.**	**PR (%)**	**No.**	**PR (%)**	**No.**	**PR (%)**
*An. funestus*	Indoors	70	84.3	69	84.1	900	89.7	1039	88.9
	Outdoors	130	86.2	146	84.9	1984	87.9	2260	87.6
	Bedroom	133	88.0	149	85.2	827	90.6	1109	89.5
	Pit shelter	178	80.3	180	88.3	487	93.2	845	89.5
*An. arabiensis*	Indoors	96	61.5	193	45.1	47	53.2	336	50.9
	Outdoors	253	59.3	727	38.5	242	59.5	1222	47.0
	Bedroom	277	81.9	58	87.9	48	83.3	383	83.0
	Pit shelter	544	73.3	493	80.9	447	80.8	1484	78.1
*An. mascarensis*	Indoors	19	*	24	*	66	89.4	109	85.3
	Outdoors	105	76.2	174	81.0	869	88.4	1148	86.1
	Bedroom	5	*	1	*	12	*	18	*
	Pit shelter	70	88.6	91	86.8	241	92.1	402	90.3

*Parous rate not calculated when No. of nuliparous + parous mosquitoes <25.

The mean parous rate of *An. funestus* was 85.6%, 86.0% and 89.4% in Andranonahoatra, Soanierana and Analamiranga, respectively (Table 6). These differences were not statistically significant. No significant differences were observed in PR of *An. funestus* collected (1) in Andranonahoatra and Soanierana compared to Analamiranga during the pre-IRS period and (2) outdoors compared to indoors during the whole study. Compared to pre-IRS period, a significant impact of IRS with DDT was observed in reducing the PR (OR = 0.47, P = 0.001), but without significant reduction of IRS with pyrethroid (OR = 0.98). Such analysis failed to test the impact of IRS on PRs of *An. arabiensis* and *An. mascarensis* due to their very low PRs and the impossibility to adjust the models.

**Table 6:** **Number of *Anopheles funestus *** **and parous rate (PR), per trimester (T) before and after indoor residual spraying (IRS) with DDT or pyrethroid (pyr)**

**Village**	**Andranonahoatra**		**Soanierana**		**Analamiranga**	
	**No.**	**PR (%)**	**No.**	**PR (%)**	**No.**	**PR (%)**
Oct 02-Feb 03 (pre-IRS)	377	86.4	241	90	629	87.5
Mar 03-May 03 (T1 DDT)	68	75	263	82	1,042	85.6
Jun 03-Aug 03 (T2 DDT)	22	95	ND	ND	460	96.5
Sept 03-Nov 03 (T3 DDT)	16	12/16*	21	76	96	82.2
Dec 03-Feb 04 (T4 DDT)	46	85	36	72	451	92.9
Mar 04-May 04 (T1 pyr)	73	90	76	92	995	86.4
Jun 04-Aug 04 (T2 pyr)	10	5/10*	24	92	499	94.9
Sept 04-Nov 04 (T3 pyr)	23	87	20	95	109	93.5
Dec 04-Feb 05 (T4 pyr)	50	94	27	89	378	92.0
Overall	685	85.6	708	86.0	4,659	89.4

ND = Not done *Parous rate not calculated when No. of nuliparous + parous mosquitoes <25.

#### Mosquito infection

**Figure 7:**
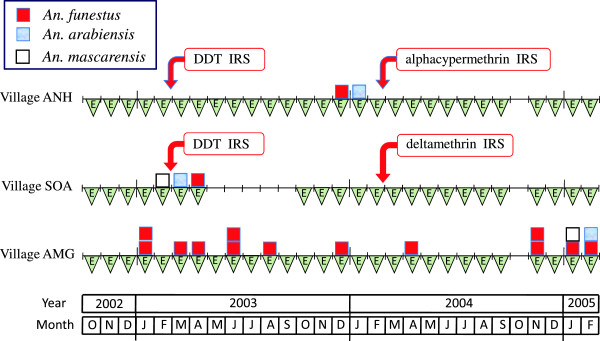
**Distribution of the 20 anophelines ELISA-CSP positive for*Plasmodium*sp., belonging to 3 anopheles species, in the three villages.** Indoor residual spray (IRS) with DDT and pyrethroids were performed in February 2003 and February 2004, respectively; the village AMG remained untreated.

A total of 20 anophelines were ELISA-CSP positive with, 15, 3 and 2 *An. funestus*, *An. arabiensis* and *An. mascarensis*, respectively (Table 7). The three species are readily involved in the *Plasmodium* transmission in the three villages, except *An. mascarensis* in Andranonahoatra. The temporal distribution of these mosquitoes is presented Figure 7. They were obtained using human landing catches (two indoors and eight outdoors), and collection of mosquitoes at rest in bedrooms (6) and in exophilic places (three in pit shelters and one in a hole). The parasite species *P. falciparum* and *P. vivax* were recorded in 12 and 8 anophelines, respectively (Table 7). In the AMG village that remained IRS untreated for the whole study, the mean sporozoitic index (SI) was 0.17%, 0.08% and 0.07% in *An. funestus*, *An. arabiensis* and *An. mascarensis*, respectively (Table 8). The mean SI for the three vectors was not significantly different between villages during the pre-IRS period, after IRS with DDT and after IRS with pyrethroids.

**Table 7:** **Number of anophelines positive for *Plasmodium *** **sp. in ELISA CSP, per village and vector species**

**Village**		**Andranonahoatra**	**Soanierana**	**Analamiranga**	**Total**
*P. falciparum*	*An. funestus*	1	1	7	13
	*An. arabiensis*	1	1	1	
	*An. mascarensis*		1		
*P. vivax*	*An. funestus*			6	7
	*An. mascarensis*			1	
Total	*An. funestus*	1	1	13	15
	*An. arabiensis*	1	1	1	3
	*An. mascarensis*		1	1	2
Grand total		2	3	15	20

**Table 8:** Infectivity rate of anophelines and [confidence interval 95%], per village and vector species

**Village**	**Andranonahoatra**	**Soanierana**	**Analamiranga**
*An. funestus*	1 / 1,273 = 0.00079 [0-0.00232]	1 / 922 = 0.00108 [0-0.00321]	13 / 7,463 = 0.00174 [0.00080-0.00269]
*An. arabiensis*	1 / 3,256 = 0.00031 [0-0.00091]	1 / 3,032 = 0.00033 [0-0.00098]	1 / 1,274 = 0.00078 [0-0.00232]
*An. mascarensis*	0 / 332 = 0	1 / 318 = 0.00314 [0-0.00930]	1 / 1,429 = 0.00070 [0-0.00207]

#### Entomological inoculation rate (EIR)

The daily EIR values per village and period underlined (1) the major contribution of *An. funestus* in the EIR, (2) the differences between villages during the pre-treatment period, and (3) the impact of IRS with pyrethroid (Table 9). Faced to the small sample of infected anophelines and to the difficulty of managing the individual variability of the biting rate, the statistical analysis of the EIR was not attempted.

**Table 9:** Daily entomological inoculation rate (EIR), per village and period before and after indoor residual spraying (IRS)

**Period**	**Village**	**Andranonahoatra**	**Soanierana**	**Analamiranga**
pre-IRS	EIR *An. funestus*	0	0	0.00422
	EIR 3 vectors*	0	0.00292	0.00422
IRS DDT	EIR *An. funestus*	0.00016	0.00145	0.00808
	EIR 3 vectors*	0.00044	0.00201	0.00808
IRS pyrethroid	EIR *An. funestus*	0	0	0.00954
	EIR 3 vectors*	0	0	0.01048

*An. funestus + *An. arabiensis* + *An. mascarensis.*

### Schoolchildren and blood parasites

The 5,174 blood smears were obtained from schoolchildren ranging 4 to 16 years old (99.3% between 6 and 12), with a sex ratio (M/F) of 0.856, and a mean axillary temperature of 37.0°C (maximum 41.4). Overall, 1,030 blood smears (19.9%) presented at least one parasite belonging to the genus *Plasmodium*. The four *Plasmodium* species infecting humans were encountered in the following proportion: *P. falciparum* 89.6%, *P. vivax* 12.5%, *P. malariae* 1.3% and *P. ovale* 0.5% (total = 103.9% due to mixed infections). Among the 1,030 schoolchildren with blood parasites, fever was present in 512 (49.7%). The mean temperature was 36.93°C in children without parasites versus 37.45°C in children with parasites. Children with both blood parasites and fever represented 9.9% of those present at school.

#### Plasmodic index

**Figure 8:**
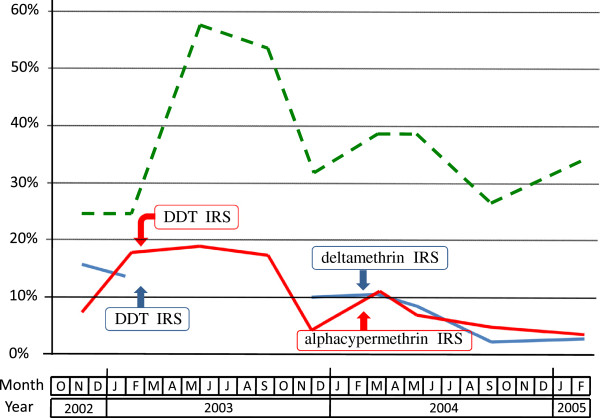
**Plasmodic index in schoolchildren in the communes of Ankadinondry-Sakay (red line), Mahasolo (blue line), and Maroharona (green dashed line).** Indoor residual spray (IRS) with DDT and pyrethroids were performed in February 2003 and February 2004, respectively; the Maroharona commune remained untreated.

For the whole study, the mean plasmodic index was 10.9%, 9.5% and 36.9% in Ankadinondry-Sakay, Mahasolo and Maroharona communes, respectively (Table 10), with important seasonal variations (Figure 8).

**Table 10:** Plasmodic index (PI) and 95% confidence interval (CI), per commune

**Commune**	**Ankadinondry-Sakay**			**Mahasolo**			**Maroharona**		
**Month**	**No.**	**PI%**	**CI 95%**	**No.**	**PI%**	**CI 95%**	**No.**	**PI%**	**CI 95%**
Nov 02	227	7.5	4-12	230	16.5	12-22	223	24.2	19-30
Feb 03	202	19.3	14-25	215	14.4	10-20	212	25.0	19-31
May 03	201	19.9	15-26	ND	ND	ND	215	57.7	51-64
Sep 03	206	18.4	13-24	ND	ND	ND	205	54.6	48-62
Nov 03	209	4.8	2-9	202	10.4	7-15	204	31.8	26-39
Mar 04	202	11.9	8-17	202	11.4	7-17	203	38.9	32-46
May 04	202	7.4	4-12	202	8.4	5-13	202	38.2	32-46
Sep 04	200	5.0	2-9	204	2.0	1-5	204	27.0	21-34
Feb 05	202	4.0	2-8	200	2.5	1-6	200	35.0	28-42
Overall	1851	10.9	9.5-12.3	1455	9.5	10.0-11.0	1868	36.9	34.7-39.1

ND = Not done.

During the pre-IRS period, this index was 13.1%, 15.5% and 24.6%, respectively. During this period and compared to the Maroharona commune without IRS, the plasmodic index was 57% and 40% lower in Ankadinondry-Sakay and Mahasolo, respectively (odds ratio (OR)=0.43, P < 0.0001 and OR = 0.60, P = 0.002), and did not differ significantly between Ankadinondry-Sakay and Mahasolo. In November 2003, compared to the pre-IRS period and controlling for the variation of the plasmodic index in Maroharona, the plasmodic index was 73% and 54% lower in Ankadinondry-Sakay and Mahasolo, respectively (OR = 0.23, P < 0.0001 and OR = 0.44, P = 0.012), without significant difference between the two communes, suggesting an impact of IRS with DDT. After IRS with pyrethroids, compared to the pre-IRS period and controlling for the variation of the plasmodic index in the commune without IRS, the reduction of the plasmodic index varied between one month and 12 months after the IRS from 66% to 92% in the commune treated by deltamethrin (OR between 0.34 and 0.08, always with P ≤ 0.001) and from 51% to 82% in the commune treated by alphacypermethrin (OR between 0.49 and 0.18, always with P ≤ 0.025), This reduction was significantly lower in Ankadinondry-Sakay than in Mahasolo (OR = 0.33 vs. OR = 0.09, P = 0.038), only in September 2004, *i.e.* 7 months after IRS with pyrethroids, suggesting a significantly longer impact of deltamethrin than alphacypermethrin.

#### Density of asexual P. falciparum

The median of log-transformed densities of asexual *P. falciparum* parasites was 329, 460 and 545 per mm^3^ of blood in Ankadinondry-Sakay, Mahasolo and Maroharona communes, respectively (Table 11). This median density was not statistically different between communes during the pre-IRS period, as for the all study duration. Similar conclusions were obtained without log transformation of densities (results not shown).

**Table 11:** **Median of densities (>0) of asexual *P. falciparum *** **(parasites/mm**^**3**^** of blood, log transformed) in schoolchildren, per commune**

**Commune**	**Ankadinondry-Sakay**			**Mahasolo**			**Maroharona**		
**Month**	**No.**	**Median**	**P25-P75**	**No.**	**Median**	**P25-P75**	**No.**	**Median**	**P25-P75**
Nov 02 and Feb 03	53	1059	433-3146	59	471	96-971	94	630	200-1243
Overall	174	329	80-1305	118	460	96-1081	590	545	110-1524

P25-P75 = Percentiles 25 and 75.

#### Malaria attacks

**Figure 9:**
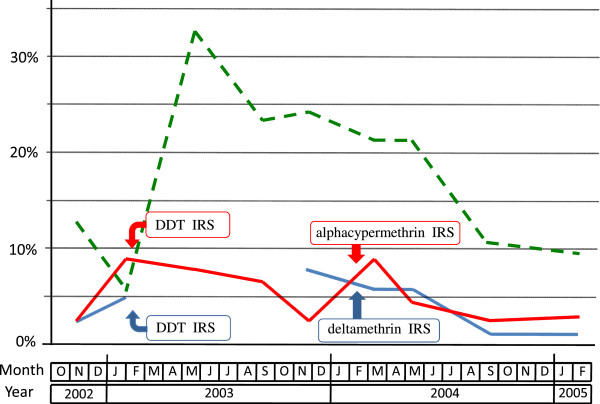
**Prevalence of malaria attack (*i.e.*fever + asexual*Plasmodium*sp.) in schoolchildren in the communes of Ankadinondry-Sakay (red line), Mahasolo (blue line), and Maroharona (green dashed line).** Indoor residual spray (IRS) with DDT and pyrethroids were performed in February 2003 and February 2004, respectively; the Maroharona commune remained untreated.

For the whole study, the mean incidence of malaria attacks was 4.0%, 4.0% and 18.0% in Ankadinondry-Sakay, Mahasolo and Maroharona communes, respectively (Table 12), with important seasonal variations (Figure 9).

**Table 12:** Malaria attacks (axillary fever >37°C plus asexual parasites in blood) of schoolchildren and 95% confidence interval (CI), per commune

**Commune**	**Ankadinondry-Sakay**			**Mahasolo**			**Maroharona**		
**Month**	**No.**	**%**	**CI 95%**	**No.**	**%**	**CI 95%**	**No.**	**%**	**CI 95%**
Nov 02	6	2.6	0.5-4.7	6	2.6	0.5-4.7	30	13.4	8.9-17.9
Feb 03	17	8.4	4.6-12.2	10	4.7	1.9-7.6	12	5.7	2.6-8.8
May 03	15	7.5	3.8-11.2	ND	ND	ND	70	32.6	26.3-38.9
Sep 03	13	6.3	3.0-9.6	ND	ND	ND	48	23.4	17.6-29.2
Nov 03	6	2.9	0.6-5.2	15	7.4	3.8-11.0	49	24.0	18.1-29.9
Mar 04	17	8.4	4.6-12.2	12	5.9	2.7-9.1	44	21.7	16.0-27.4
May 04	9	4.4	1.6-7.2	12	5.9	2.7-9.1	43	21.3	15.7-27.0
Sep 04	5	2.5	0.3-4.7	2	1.0	0-2.4	22	10.8	6.5-15.1
Feb 05	6	3.0	0.6-5.4	2	1.0	0-2.5	19	9.5	5.4-13.6
Overall	94	4.0	3.1-4.9	59	4.0	3.0-5.0	337	18.0	16.3-19.7

ND = Not done.

During the pre-IRS period, the mean prevalence of clinical malaria attacks was 4.0%, 4.0% and 18.0% in Ankadinondry-Sakay, Mahasolo and Maroharona communes, respectively. During this period and compared to the Maroharona commune without IRS, the mean prevalence of clinical malaria attacks was 64% and 49% lower in Ankadinondry-Sakay and Mahasolo, respectively (OR = 0.36, P < 0.0001 and OR = 0.51, P = 0.007), and did not differ significantly between Ankadinondry-Sakay and Mahasolo. In November 2003, compared to the pre-IRS period and controlling for the variation of the plasmodic index in Maroharona, the reduction of the prevalence of clinical malaria attacks was 82% in Ankadinondry-Sakay (OR = 0.18, P = 0.001), although this reduction was not significant in Mahasolo (OR = 0.73). After IRS with pyrethroids, compared to the pre-IRS period and controlling for the variation of the plasmodic index in Maroharona, the reduction of the prevalence of clinical malaria attacks did not differ significantly between Ankadinondry-Sakay and Mahasolo, and varied between one month and 12 months after the IRS from 36% (OR = 0.64, P = 0.193) to 56% (OR = 0.44, P = 0.094) with maximum reductions of 54% (OR = 0.46, P = 0.035) and 67% (OR = 0.33, P = 0.028) observed three and seven months after IRS, respectively, suggesting a significant and similar impact of deltamethrin and alphacypermethrin. Similar conclusions were obtained when a threshold of 500 or 1,000 asexual parasites per ml of blood was introduced to define a malaria attack (results not shown).

## Discussion

The present survey combines entomological and parasitological studies from the Malagasy Highlands and demonstrates the efficiency of IRS with DDT and IRS with pyrethroids (alphacypermethrin and deltamethrin) by analysing pre-and post-treatment data of two villages compared to a control village without intervention.

The entomological observations show the three anopheline species involved in the transmission of malaria on the Malagasy Central Highlands. *An. funestus*, *An. arabiensis* and *An. mascarensis* were found infected. The prominent role of *An. funestus* as vector of malaria parasites is highlighted. This species exhibits an intermediate behaviour endo-exophilic behaviour both for biting activity and resting. The indoor spraying with insecticide impacts its vectorial capacity. These observations claim for the sensitivity of *An. funestus* to used insecticides in the Central Malagasy highlands. *An. arabiensis* and *An. mascarensis* are secondary vectors and typically exophilic; indeed, most of those collected in this study were positive for zebu cattle blood. Consequently these anophelines are relatively less impacted by IRS. The degree in exophily appears linked to the degree in zoophily. However, the moderate impact of IRS with insecticide on the secondary vectors, if not ideal, does not render this vector control strategy ineffective as a great reduction in malaria transmission after IRS shows. Although, even with correct implementation, IRS with insecticide did not eliminate malaria transmission. This has been observed on many similar occasions [4,11,19,20,26,32]. The persistence of malaria vectors, with or without malaria cases, following an effective vector control measure is a known phenomenon since the 1960s [48].

The parasitological observations illustrate the prominent infection with *P. falciparum* in schoolchildren. In addition, three other malarial species are also prevalent, especially *P. vivax*. Despite a relatively low level of malaria transmission (a daily EIR = 0.009, as observed in the untreated village corresponds to an annual EIR = 3.3), positive parasitaemia was detected in schoolchildren suffering from uncomplicated simple malaria attacks. IRS with insecticide is rapidly followed by a reduction in prevalence of blood parasites and malaria attacks, both positive consequences. This has also been found elsewhere [8,33,34,49].

These conclusions are reasonably robust. But some points can be discussed.

The three studied communes are similar in surface area, climate, altitudinal elevation, human ethnic group, population density and agricultural activities. They also share a common border. In addition, the three studied villages are alike for human and zebu cattle population size, habitat, distance to running water, and elevation with regards to piedmont and rice field of sandbank. However noticeable differences were observed concerning entomological aspects such as relative proportions of anopheline species, unexplained increase in density (*e.g. An. funestus* in Soanierana but not in the two other villages the month following IRS with DDT) or decrease (*e.g. An. mascarensis* in the untreated Analamiranga in July-September 2004), and parasitological observations (*e.g.* the evolution of parasite ratio in the pre-treatment period in Andranonahoatra versus Soanierana). This set of uncontrolled inter-variations arising between zones and proper environmental factors [28] limit comparisons between villages/communes. This constitutes an *a posteriori* confirmation of the relevance of our data processing which highlighted the evaluation before versus after the implementation of the control measures rather than an evaluation between communes/villages.

As already stressed, the impact of IRS with insecticide is more effective on the endo-exophagic *An. funestus* than the exophagic *An. arabiensis*. Blood feeding behaviour and departure from houses were studied in Kenya under the impact of permethrin-impregnated eaves-sisal curtains: *An. funestus* shifted to feeding more on cattle [50]. According to the present study, in Ethiopia, 1.5% of outdoor-resting *An. arabiensis* and 66% of those collected indoors had fed on humans, and human baits outdoors caught > 2.5 times more *An. arabiensis* than those indoors [51].

– The general tendency for reduction of parasite prevalence in schoolchildren throughout the study was significant and expected. On the other hand a reduction of the parasite prevalence in the untreated commune of Maroharona remains partly unexplained; this may be a secondary effect of the presence/activity of the medical team in charge of the present study who provided anti-malarial drugs for treatment in cases of suspected malaria, in agreement with the national health policy.

– In the present study, 17 anophelines were detected positive with *P. malariae* and 13 with *P. falciparum* (Additional file 2: Table S2). In comparison, prevalences of these plasmodial species in children were 1.3% and 89.6%, respectively; when looking at gametocyte prevalence the percentages were 0.6% [0.2%-1.3%] and 14.7% [CI 95% = 12.6%-17.0%] (Additional file 1: Table S1). These observations are puzzling and question the true *P. malariae* infection of mosquitoes*.* The detection of infected mosquitoes was here performed using the ELISA, a technique known to produce false positives in the detection of circumsporozoite protein (CSP) in mosquitoes consequently of overestimating the infectivity rate [52-54]. Whatever is the cause of such overestimation, results on *P. malariae* infectivity of mosquitoes were not considered in the present study. Both infectivity rate and EIR were calculated on *P. falciparum* and *P. vivax*, two species that totalized 99% of *Plasmodium* infection in child population.

– By study design, the schoolchildren not present at school the day of the survey were not considered in the study. One may presume that a lot of sick children does not reach the school. On the contrary, because the date of the survey was announced in advance, the sick children went to school, sure to receive a free anti-malarial treatment. In other words, it is hard to know if the reported prevalence of malaria attacks in children at school is underestimated or not.

– Three insecticides were used, DDT, alphacypermethrin and deltamethrin. Overall, similar efficacies were recorded and thus each one can be applied indistinctly for malaria vector control in the highlands. Many entomological and parasitological variables were lower after IRS with pyrethroid than after IRS with DDT. This cannot be interpreted as a lower efficacy of DDT compared to pyrethroid for two reasons. First, the pyrethroids were sprayed 12 months after DDT, in a time when the persistent effect of DDT was still partly effective on the house walls [49]. That is to say pyrethroids were probably not tested alone but with a small additive effect of DDT. Second, the reduction of plasmodic index and incidence of malaria attacks in 2004-05 in the untreated area impede to conclude for a better efficacy of pyrethroid by regard to DDT. Nevertheless the use of DDT was recently in the topic of discussion, especially when used in IRS in Africa [10-12].

Highlights can be raised for the future. Infected mosquitoes were collected in outside shelters, notably in the pit shelters that can be considered as the most 'outdoors’ resting place examined. Insecticide spraying with insecticides outside houses could be useful, especially during malaria outbreaks or in the objective of the malaria eradication in Madagascar. Additionally, as the risk of re-invasion of vectors from untreated areas to treated areas is high [55,56], the level of IRS coverage is an important key points for efficient IRS.

Finally, this research project points out the fact that vector control per se will not reach achievement of elimination in area where the vectors exhibit a large spectrum of endo/exophilic behaviours in response to insecticide pressure. In order to reach the goal of pre-elimination there is also a need for rapid diagnostic of malaria parasites in humans and effective treatment. Such improvements are required for the next step of passing from pre-elimination to elimination phase in area where transmission still occurs. A transmission blocking vaccine could be one the needed new tools.

## Conclusions

The results presented here suggest that a significant reduction of man-anopheline contact is possible, especially for *An. funestus*, the main vector in the Malagasy Highlands. In this region, the entomological inoculation rate is relatively low, about 3.3 bites of infected anophelines per man per year in the untreated village. The reduction of malaria transmission after IRS with insecticide, without additional control measures (like a better prevention, diagnostic, treatment, health services, etc.), is associated with a significant decrease in the prevalence of blood parasites and of malaria attacks in children. The reduction of risk was equivalent between IRS using pyrethroids and DDT, especially on vector biting and resting behaviour inside houses. The present study also shows that pyrethroids (alphacypermethrin and deltamethrin) are efficient alternatives to DDT when used in indoor residual spraying in the Highlands of Madagascar.

## Competing interests

The authors declare that they have no competing interests.

## Authors’ contribution

JR participated in the study design, data collection and data analysis; MER participated in the study design and supervised the IRS; MR and VaRa participated in the parasitological study; LA and GLG participated in the entomological study; CR and SB performed the statistical analysis. FA and ViRo conceived and coordinated the study. All authors participated in drafting the manuscript and approved the final manuscript.

## Supplementary Material

Additional file 1: Table S1 MahasoloParasito 2002–2005.xls. Crude demographic, parasitological and clinical data collected during the whole study. Each of the 5,174 lines concerns one schoolchild in the sheet 1. The 34 columns are detailed in the sheet 2. Click here for file

Additional file 2: Table S2 MahasoloEntomo 2002-2005.xls. Crude entomological data for the three malaria vectors collected during the whole study. Each of the 19,789 lines concerns one anopheline vector in the sheet 1. The 15 columns are detained in the sheet 2. The sampling effort is detailed in the sheet 3. Click here for file
